# Benchmark-Based Evaluation of ChatGPT and Gemini in Radiation Oncology: Performance, Limitations, and Challenges for Clinical Interpretation

**DOI:** 10.7759/cureus.112361

**Published:** 2026-07-09

**Authors:** Yousef Mohammed, Ronny Kruschel, Nizar Alshammas, Klaus Pietschmann

**Affiliations:** 1 Radiation Oncology and Radiotherapy, SRH Zentralklinikum Suhl, Suhl, DEU; 2 Radiotherapy and Radiation Oncology, Jena University Hospital, Jena, DEU; 3 Radiotherapy and Radiation Oncology, SRH Zentralklinikum Suhl, Suhl, DEU; 4 Radiation Oncology, Helios Medical Care Centers GmbH, Aue, DEU

**Keywords:** artificial intelligence, chatgpt, clinical decision support, gemini, large language models, radiation oncology

## Abstract

Background

Large language models (LLMs) are increasingly being explored for medical applications, including clinical decision support and oncology education. However, their performance in radiation oncology remains insufficiently characterized.

Methods

This study evaluated and compared the performance of ChatGPT (GPT-5.2; OpenAI, San Francisco, USA) and Gemini (Ultra/Pro; Google, Mountain View, USA) in radiation oncology. A benchmark consisting of 70 multiple-choice questions covering clinical oncology, radiation physics, and radiobiology was used to assess general knowledge. In addition, 25 clinically relevant open-ended questions were independently evaluated by three radiation oncologists using five-point Likert scales for correctness and usefulness. A mixed-effects model was applied to analyze performance.

Results

ChatGPT achieved an accuracy of 94.3%, while Gemini achieved 97.1% in the multiple-choice assessment. For open-ended questions, both models received similarly high ratings, with mean correctness scores of 4.71 and 4.67 and mean usefulness scores of 4.63 and 4.64 for ChatGPT and Gemini, respectively. Mixed-effects analysis demonstrated a significant effect of question type on both correctness and usefulness, whereas no significant differences between models were observed. Although most responses were rated as good or very good, limitations became apparent in more complex clinical scenarios requiring prioritization and individualized decision-making. Minor discrepancies between benchmark answers and current clinical evidence were also identified.

Conclusions

Both ChatGPT and Gemini demonstrated high performance on benchmark-based radiation oncology assessments. While the generated responses were generally accurate, limitations remained in complex clinical scenarios requiring nuanced clinical judgment. Further studies are needed to determine whether such performance translates into meaningful clinical utility in real-world radiation oncology practice.

## Introduction

Large language models (LLMs) have rapidly evolved from experimental research tools into accessible systems with growing relevance in medicine. They are increasingly being explored across multiple domains, including medical education, clinical documentation, access to medical information, and decision support. In particular, LLMs have shown potential in assisting with diagnostic tasks, answering medical questions, and supporting clinical workflows, although their integration into routine clinical practice remains limited by important challenges such as accuracy, bias, and the need for careful validation [1-3]. Radiation oncology represents a complex clinical domain that requires a multidisciplinary approach, continuous integration of evolving evidence, and the combination of clinical judgment with radiobiological and physical principles. Treatment decisions often require an individualized approach based on tumor characteristics, patient-related factors, and guideline-based recommendations. Given this complexity, LLMs are increasingly being explored as potential tools to support clinical decision-making and workflow processes in oncology. While early studies suggest that LLMs can assist in generating treatment-related information and processing complex clinical data, their application in oncology remains limited and requires careful validation, expert oversight, and domain-specific adaptation to ensure safe and effective use [4,5]. Previous studies have explored the possibility of implementing LLMs in radiation oncology [6,7]. For example, Dennstädt et al. demonstrated that earlier-generation models such as ChatGPT-3.5 (OpenAI, San Francisco, USA) were able to answer a substantial percentage of multiple-choice and open-ended radiation oncology-related questions, albeit with notable limitations. Since then, LLM technology has advanced considerably, with newer LLM generations with higher performance across a wide range of tasks becoming more available [8,9]. As LLMs' performance improves, it is important to test the reliability of the LLMs. In this context, evaluating current-generation models, comparing different systems, and identifying persistent limitations despite high apparent performance are of particular relevance. Therefore, the aim of this study was not only to compare the performance of two contemporary LLMs in radiation oncology but also to explore the strengths and limitations of benchmark-based evaluation approaches, including challenges related to clinical interpretation, reference-answer validity, and assessment of open-ended responses.

## Materials and methods

L‌LM models

In this study, two contemporary LLMs were evaluated between 1 January and 30 January 2026: ChatGPT (GPT-5.2; OpenAI, San Francisco, USA) and Gemini (Ultra/Pro; Google, Mountain View, USA). ChatGPT (GPT-5.2) was accessed through the paid ChatGPT Plus subscription and represented the version available at the time of testing. Gemini (Ultra/Pro), accessed through Google's paid subscription service, is an advanced large language model designed for complex reasoning tasks across multiple domains, including medicine. Both models were queried under standardized conditions using identical input prompts.

Evaluation

The evaluation framework was based on a previously established benchmark in radiation oncology as described by Dennstädt et al. [6]. The study consisted of two complementary components designed to assess both factual knowledge and clinical reasoning capabilities of the evaluated models. For the assessment of general knowledge, a set of 70 multiple-choice questions was used. Each question provided four possible answer options (A-D), with only one correct answer. The questions covered three domains: clinical radiation oncology (n=44), radiation physics (n=14), and radiobiology (n=12). All questions were submitted in English to both models using their respective web-based interfaces. Each question included the instruction to provide only a single answer choice and was entered in a new session. All responses were recorded without modification. The benchmark answer key was used as originally published; however, selected items were critically reviewed against current clinical guidelines and literature.

To evaluate performance in clinically relevant scenarios, a set of 25 open-ended questions was used. These questions addressed key aspects of radiation oncology, including patient evaluation, treatment indication, treatment planning, plan evaluation, and treatment-related toxicity. Model-generated responses were lightly preprocessed before evaluation to remove non-medical conversational elements (e.g., introductory phrases, formatting-related headings), while preserving all clinically relevant content. The open-ended responses were evaluated by three radiation oncologists using a structured assessment form, with a median of 13 years of clinical experience in radiation oncology (range: 5-18.5 years). Evaluators were aware that the responses were generated by large language models but were blinded to the specific model attribution. Evaluators were instructed to assess the responses solely based on their medical content.

Correctness and usefulness were assessed independently using a five-point Likert scale (1=very bad, 5=very good), following the general evaluation approach described by Dennstädt et al. [6]. Evaluators were asked to base their judgments on generally accepted medical standards and to avoid reliance on individual clinical preferences. Despite the limited number of raters, ratings were generally similar across evaluators, with only minor variations observed for most items. In addition, evaluators were invited to provide free-text comments and suggestions for improvement. When necessary, they were allowed to consult relevant medical literature to support their evaluations.

The complete set of multiple-choice questions (questions 1-70, Appendix S1) and the open-ended clinical questions (questions 1-25, Appendix S2) can be accessed through the Google Drive link provided in the Appendix.

Statistical analysis of open-ended responses

A mixed-effects model was used for data analysis, with correctness and usefulness scores as dependent variables, question and model as fixed factors, and rater as a random effect. Data analysis was performed using Jamovi Version 2.3.28.0 (The jamovi Project, Sydney, Australia).

## Results

Performance on multiple-choice questions (1-70)

**Figure 1:**
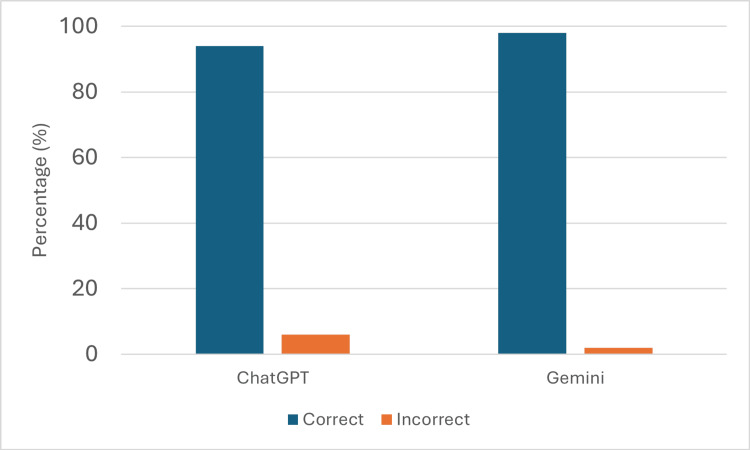
Performance of ChatGPT and Gemini on multiple-choice questions (n=70).

ChatGPT correctly answered 66 of 70 multiple-choice questions (94.3%), while Gemini achieved 68 correct responses (97.1%). Both models demonstrated a high level of accuracy across all domains (Figure 1). Two questions were answered incorrectly by both models. These included a question based on the Preoperative Chemotherapy or Radiochemotherapy in Esophagogastric Adenocarcinoma (POET) trial on neoadjuvant chemoradiotherapy for gastroesophageal junction cancer (question 31), as well as the International Federation of Gynecology and Obstetrics (FIGO) staging question for vulvar cancer (question 35). In both cases, the responses provided by the models differed from the original benchmark key. In two additional questions, ChatGPT provided incorrect answers while Gemini answered correctly. These included a tumor, node, metastasis (TNM) staging question for vulvar cancer (question 34) and a physics-related dosimetry question (question 54). No questions were answered incorrectly by Gemini alone. Most incorrect responses occurred in the clinical domain, followed by a single incorrect answer in radiation physics, while all radiobiology questions were answered correctly by both models.

Evaluation of open-ended responses (1-25)

**Figure 2:**
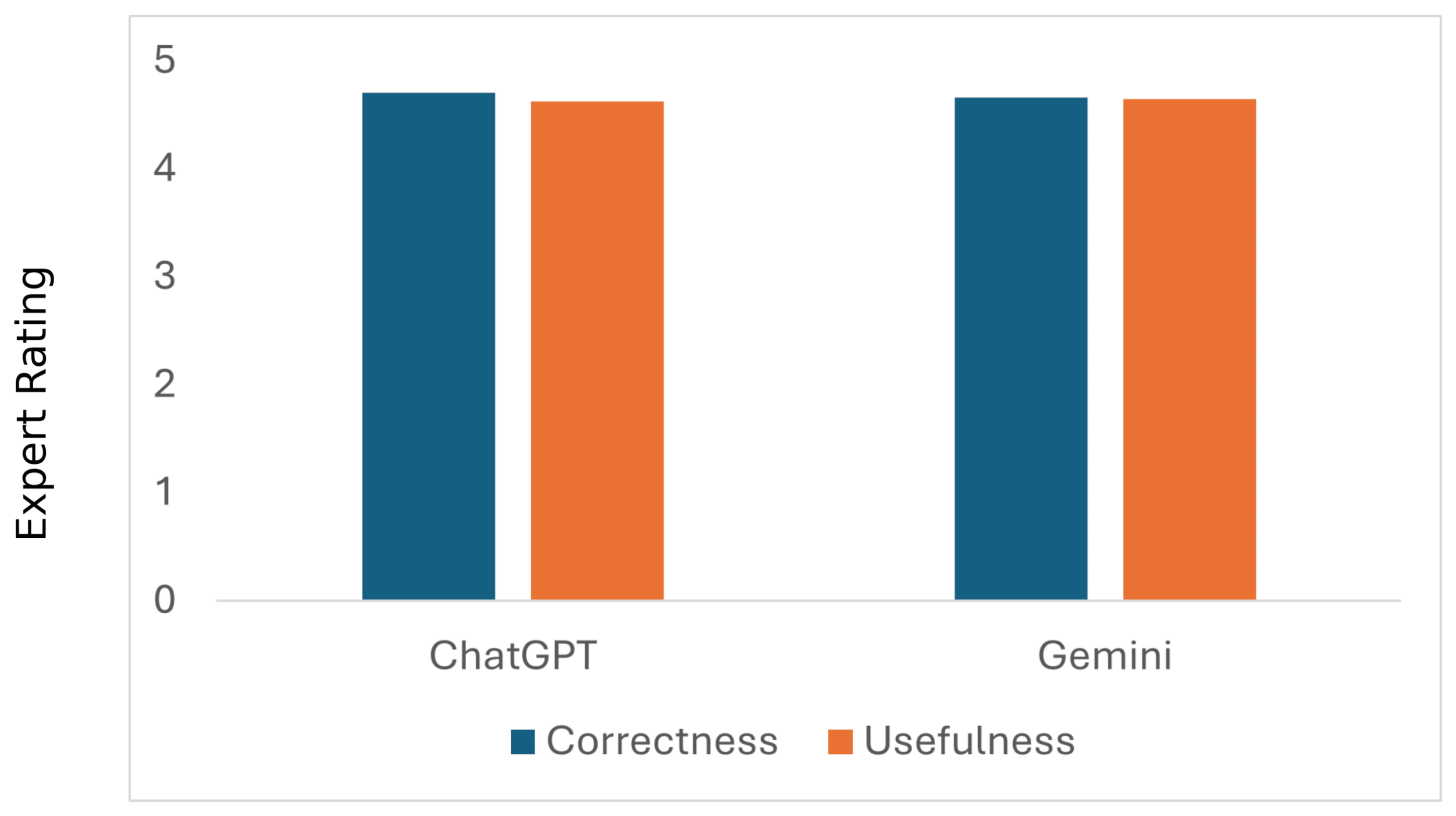
Evaluation of correctness and usefulness scores for ChatGPT and Gemini based on expert assessment of open-ended questions.

Overall, the evaluation of open-ended responses showed consistently high ratings across all questions (Figure 2). Most responses were assessed as good or very good (scores 4-5) by all three evaluators. Mean correctness scores were 4.71 for ChatGPT and 4.67 for Gemini (both range, 3-5), while mean usefulness scores were 4.63 and 4.64, respectively (both range, 3-5). In four of 25 questions, at least one evaluator assigned a score of 3 for correctness and/or usefulness. These lower ratings were observed in the prostate cancer case (question 7), a question on early-stage anal carcinoma (question 2), a question on adjuvant axillary radiotherapy in breast cancer (question 3), and a clinical scenario involving a patient with poor performance status (question 4). In these cases, the responses demonstrated minor inaccuracies or imprecise clinical details. In more complex clinical scenarios, lower scores were primarily related to aspects of clinical decision-making.

Mixed-effects analysis of open-ended response performance

There was a significant effect of the type of question on both the correctness (F=11.43; p<0.001) and the usefulness (F=12.2; p=0.002). The type of model that was used did not have a significant impact on correctness (F=1.22; p=0.272) or usefulness (F=0.17; p=0.678), suggesting that both models are performing similarly.

## Discussion

Principal findings and interpretation

In the multiple-choice assessment, an important observation in our analysis was the presence of discrepancies between the benchmark answer key and current clinical evidence. In selected multiple-choice questions, both models provided answers that differed from the benchmark but were consistent with contemporary literature. For example, in a question based on the POET trial (question 31), which evaluated the impact of neoadjuvant chemoradiotherapy versus chemotherapy alone on pathological response and overall survival in gastroesophageal junction adenocarcinoma, the benchmark designated option A as correct, indicating that neoadjuvant chemoradiotherapy provided an advantage in both pathological complete remission and overall survival. However, both LLMs selected option B. While the trial demonstrated a statistically significant improvement in pathological complete remission (15.6% vs. 2.0%, p=0.03), the overall survival difference did not reach statistical significance [three-year overall survival (OS) 47.4% vs. 27.7%, p=0.07] due to premature trial closure [10]. Notably, the long-term follow-up of the POET trial confirmed that overall survival remained statistically nonsignificant even with extended follow-up [five-year OS 39.5% vs. 24.4%, p=0.055; hazard ratio (HR) 0.65, 95% confidence interval (CI) 0.42 - 1.01] [11]. The authors themselves acknowledged that the trial "could not provide statistical significance," yet interpreted the results as pointing toward a survival advantage for chemoradiotherapy. The original benchmark appears to have accepted the authors' clinical interpretation as the correct answer, whereas the LLMs applied a stricter statistical framework in which a non-significant p-value does not constitute a proven advantage. Both interpretations are defensible, but this discrepancy underscores the importance of precise question phrasing in medical LLM benchmarks and the need for transparent answer justification in benchmark datasets.

In a question on FIGO staging of vulvar cancer (question 35), which described a localized tumor with lymph node metastasis, both models correctly classified the disease as stage III, whereas the benchmark indicated a lower stage. According to the current FIGO classification, the presence of nodal involvement upstages the disease to at least stage III, supporting the model responses [12]. A related vulvar cancer staging question (question 34) asked for TNM classification according to the American Joint Committee on Cancer (AJCC) 8th edition for the same clinical scenario (tumor confined to the vulva, 2.3 cm, stromal invasion of 1 mm, one lymph node metastasis of 4 mm). The T-stage classification depends on which AJCC TNM definitions are applied: the standard AJCC 8th edition TNM tables (aligned with FIGO 2009) define T1 as ≤2 cm and T2 as >2 cm, classifying this tumor as T2 [13], whereas an alternative AJCC interpretation includes tumors >2 cm within T1b [14]. Regardless of the T classification, the N classification is unambiguously N1a (1-2 metastases each <5 mm), not N1b (one metastasis ≥5 mm) [13]. The benchmark answer of T1N1b (answer B) is therefore incorrect on the N component. ChatGPT selected T1N1a (answer A), correctly identifying the N1a subcategory, while Gemini selected T1N1b (answer B), matching the benchmark's designated answer but incorrectly classifying the N component. Together, these two vulvar cancer staging questions illustrate a critical challenge in LLM evaluation; when benchmark answers are not fully aligned with current classification systems, models that reflect contemporary clinical standards may appear incorrect, while those matching the benchmark are scored as correct.

In the evaluation of open-ended responses, lower ratings were primarily associated with minor factual inaccuracies and limitations in clinical prioritization. For example, in a prostate cancer case (question 7), a minor inconsistency in the interpretation of postoperative prostate-specific antigen (PSA) dynamics was observed, as the response suggested a nadir below 0.1 ng/ml despite PSA values remaining above this level. In another case (question 2), imprecision regarding the timing of response assessment after chemoradiotherapy for anal cancer reflected a well-documented ambiguity between trial-based evidence and guideline recommendations. While the Anal Cancer Trial II (ACT II) trial assessed complete clinical response at 26 weeks from the start of treatment [15], clinical guidelines typically define assessment intervals from the completion of chemoradiotherapy; a difference of approximately five to six weeks given standard chemoradiotherapy (CRT) duration, which can lead to clinically meaningful differences in interpretation [16].

In a breast cancer scenario (question 3), which asked for the indications for adjuvant axillary radiotherapy, both answers provide generally accurate overviews but occasionally overstate the strength of recommendations, particularly for 1-3 positive nodes and post-mastectomy scenarios, where current practice increasingly favors individualized, risk-adapted decisions rather than routine axillary radiotherapy [17,18]. 

In a locally advanced non-small cell lung cancer (NSCLC) scenario (question 4), which asked for the most appropriate diagnostic and treatment procedures in a patient with poor performance status (Karnofsky 60), both answers provided comprehensive and structured workup recommendations and correctly noted that concurrent chemoradiotherapy is generally not feasible at this performance level, favoring sequential therapy or radiotherapy alone. However, both responses tended to emphasize tumor-directed treatment strategies without sufficiently prioritizing the fundamental question of treatment feasibility, reflecting a limitation in clinical prioritization rather than a lack of relevant content. Current guidelines recommend that the critical first step in such patients is determining whether poor performance status is tumor-related and potentially reversible versus comorbidity driven, as this distinction dictates whether any curative intent therapy is appropriate [19,20]. These patterns may suggest that LLMs favor exhaustive therapeutic algorithms over the patient-centered clinical reasoning that characterizes real-world tumor board discussions, where factors such as frailty, quality of life, and realistic prognosis often take precedence.

Comparison with existing literature

Our findings are consistent with recent studies evaluating LLM performance in radiation oncology and related domains. For example, on standardized multiple-choice assessments such as the American College of Radiology Radiation Oncology In-Training Examination (TXIT), GPT-5 achieved a mean accuracy of 92.8%, outperforming earlier model generations [9]. Similarly, ChatGPT-5 Pro has been reported to reach accuracy rates above 90% across Japanese radiotherapy certification examinations [21]. These findings are further supported by studies evaluating LLM performance in patient-centered oncology queries. For example, ChatGPT demonstrated comparable or superior performance in the majority of radiation oncology patient questions, particularly in terms of correctness (94%) and conciseness (91%), although its responses were written at a higher reading level than expert answers, and a small number of responses (2/115) were flagged for potential harm [22]. In addition, large language models such as Llama3-OpenBioLLM-70B can answer real-world clinical questions in radiation oncology with a quality comparable to experienced clinicians, as reflected by similar Likert-based ratings [23].

Furthermore, recent studies demonstrate that large language models achieve high performance in oncology-specific tasks, with GPT-4 and Claude Opus reaching accuracies of approximately 80-86% on breast oncology multiple-choice questions, although performance may vary depending on prompting strategies such as chain-of-thought, and common errors include reliance on outdated guidelines and misinterpretation of clinical trial data [24].

Despite these advances, important limitations remain. Even in studies demonstrating high overall accuracy, errors continue to cluster in complex clinical scenarios requiring precise interpretation of trial data or individualized treatment decisions, with hallucinations still flagged in approximately 10% of expert-reviewed responses [9]. Similarly, even in models achieving clinician comparable quality, 16% of answers to real-world radiation oncology questions were deemed potentially harmful [23].

Beyond these performance-related limitations, recent evidence highlights important safety concerns. Large language models are highly susceptible to prompt-injection attacks, in which manipulated inputs can lead to unsafe or clinically inappropriate recommendations. Notably, even advanced models demonstrated substantial vulnerability in controlled simulations, indicating that high performance does not necessarily guarantee safe behavior. These findings underscore the need for robust safeguards and careful system-level integration when applying LLMs in clinical settings [25].

Strategies for improvement

**Figure 3:**
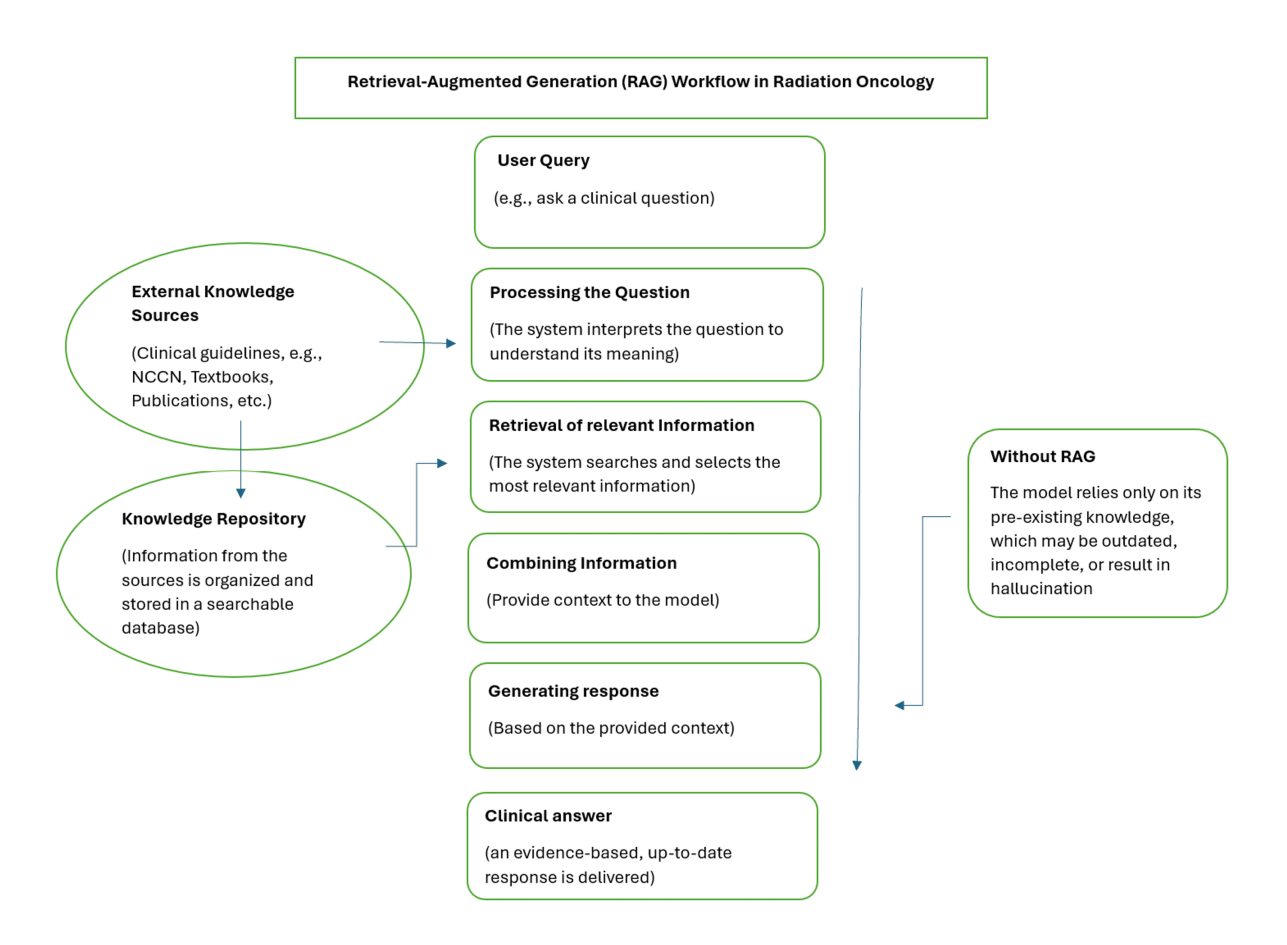
Shows a schematic overview of retrieval-augmented generation (RAG) in radiation oncology.

There is growing recognition that domain-specific adaptation strategies can further enhance the performance of LLMs in radiation oncology. In particular, fine-tuning approaches have been shown to improve model performance across several clinically relevant tasks, with a considerable proportion of generated treatment recommendations judged as clinically acceptable [26]. In parallel, retrieval-augmented generation (RAG) has been proposed as a complementary strategy, allowing models to incorporate domain-specific information from external knowledge sources at the time of inference (Figure 3). Recent data demonstrate that RAG can improve performance, particularly for models without advanced reasoning capabilities, by providing more context-specific and evidence-based responses [27]. In contrast to fine-tuning, RAG-based approaches offer greater flexibility, as they can be continuously updated with current guidelines and literature without requiring retraining of the underlying model. Together, these approaches may help address some of the limitations observed in our study, especially with regard to individualized clinical decision-making and contextual accuracy. More broadly, these developments point toward a shift from general-purpose LLMs toward increasingly specialized and adaptable AI systems tailored to radiation oncology workflows.

Clinical implications and potential applications

The results of this study suggest that LLMs may support radiation oncology workflows, particularly in medical education, knowledge retrieval, and information synthesis. However, consistent with the European Society for Medical Oncology (ESMO) guidance, they should be used as supportive tools under appropriate clinical supervision rather than as independent decision-making systems. In particular, the ESMO framework highlights the importance of human oversight, data protection, and careful validation before clinical implementation [5]. Beyond this, recent developments suggest that LLMs may also contribute to technical aspects of radiation oncology workflows. For example, visual language models that integrate imaging data with clinical text information have been shown to improve the accuracy of automated target volume delineation compared to conventional approaches [28]. In addition, recent studies suggest that LLMs may support radiotherapy treatment planning by optimizing dose parameters and generating plans with quality comparable to those created by medical physicists, while reducing planning time [29]. Taken together, these findings suggest that the role of LLMs in radiation oncology is likely to expand beyond knowledge-based support toward more integrated applications within clinical workflows. In the future, LLMs may contribute to hybrid systems that combine clinical data, imaging, and treatment planning, enabling more efficient and standardized processes across different stages of care.

Study limitations

Several limitations should be considered when interpreting the findings of this study. First, the expert evaluation was based on three radiation oncologists. Although this enabled detailed assessment by experienced clinicians, some degree of subjectivity is inherent in physician-based evaluations. Furthermore, evaluators were aware that the responses originated from LLMs, which may have introduced expectation bias. Inter-rater agreement was assessed descriptively rather than through formal reliability statistics, as ratings were consistently high with limited score variability. Second, advanced prompt-engineering strategies were not systematically explored; alternative prompting approaches may have affected the quality and clinical relevance of the generated responses. Third, the study relied on a predefined benchmark dataset that may not fully capture the complexity and diversity of real-world clinical decision-making. In addition, because the benchmark questions were publicly available, prior exposure of the models to similar material during training cannot be excluded, potentially influencing performance estimates. Finally, given the rapid evolution of LLM technology, the findings should be regarded as a time-specific assessment of model performance at the time of evaluation.

## Conclusions

Both ChatGPT and Gemini demonstrated high performance on benchmark-based radiation oncology assessments and generated largely accurate and clinically relevant responses. However, benchmark performance alone should not be interpreted as evidence of clinical utility or readiness for clinical implementation. The findings highlight both the capabilities of contemporary LLMs and the challenges associated with evaluating these systems using predefined question sets. Further studies in real-world clinical environments are required to determine their practical value and limitations in radiation oncology practice.

## Appendices

The complete set of multiple-choice questions (questions 1-70, Appendix S1) and the open-ended clinical questions (questions 1-25, Appendix S2) can be accessed through https://drive.google.com/drive/folders/17OYc8PzDS-ziKT2NFP-vtciDP_RzBLlf?usp=sharing.
